# Plant-based diet quality, fat mass, and cardiovascular disease: A mediation analysis of mid-aged adults in the UK Biobank

**DOI:** 10.1038/s41430-026-01731-4

**Published:** 2026-04-01

**Authors:** Laura E. Marchese, Sarah A. McNaughton, Gilly A. Hendrie, Barbara Brayner, Kacie M. Dickinson, Katherine M. Livingstone

**Affiliations:** 1 Institute for Physical Activity and Nutrition (IPAN), School of Exercise and Nutrition Sciences, Deakin University, Geelong, VIC 3220 Australia; 2 Health and Well-Being Centre for Research Innovation, School of Human Movement and Nutrition Sciences, University of Queensland, St Lucia, QLD 4067 Australia; 3 School of Exercise and Nutrition Sciences, Deakin University, Geelong, VIC 3220 Australia; 4 CSIRO Health and Biosecurity, Adelaide, SA 5000 Australia; 5 Caring Futures Institute, College of Nursing and Health Sciences, Flinders University, Adelaide, SA 5001 Australia

**Keywords:** Nutrition, Cardiovascular diseases

## Abstract

**Background/objectives:**

Evidence supports plant-based diets for preventing cardiovascular diseases (CVD). Fat mass is a strong predictor of CVD, however it is unclear whether this mediates the relationship between plant-based diets and CVD. Thus, this study aimed to determine if longitudinal associations between plant-based diet quality indices and incidence of CVD events, CVD mortality and all-cause mortality were mediated by fat mass in mid-aged adults.

**Subjects/methods:**

Dietary data (Oxford WebQ) from 14,233 adults from the UK Biobank were used to calculate adherence to three plant-based diet quality indices: an overall plant-based diet (PDI), a healthy plant-based diet (hPDI), and a less healthy plant-based diet (uPDI). Dual X-ray absorptiometry measured percentage fat mass. Cox proportional hazard ratios (95% CI) identified associations between the indices and CVD events, CVD mortality or all-cause mortality, and mediation analyses determined overall, direct, and indirect effects of fat mass.

**Results:**

The PDI and hPDI were inversely associated with fat mass, and the uPDI was positively associated (*p* < 0.001). In several models, fat mass was associated with CVD events, CVD mortality, and all-cause mortality after controlling for the plant-based diet quality indices. No association was found between the indices and health outcomes, with (direct effect) or without (total effect) the fat mass mediator (*p* ≥ 0.1). In several models there was a significant negative indirect effect for the PDI or hPDI on CVD events or CVD mortality via the fat mass mediator, and a significant positive indirect effect for the uPDI on CVD events and CVD mortality.

**Conclusions:**

No evidence was found for an association between the plant-based diet quality indices on the outcomes without, as well as after accounting for fat mass. However, the significant indirect effects found suggest that fat mass may be a key mechanism linking plant-based diets to CVD.

## Introduction

Cardiovascular disease (CVD) is the leading cause of death worldwide [1, 2], responsible for one third of all deaths [3], with most CVD events and deaths occurring in mid-age [4]. The risk of developing CVD is influenced by both modifiable and non-modifiable risk factors, of which diet and adiposity are among the leading modifiable risk factors in mid-life [2]. A healthy diet has been identified as an effective strategy for the prevention of CVD [5]. However, previous research has focused on individual nutrients or foods, such as increasing oily fish or reducing processed meat. This approach overlooks the impact of the overall quality of the diet, and hence there has been a recent shift to assessing whole dietary patterns [5, 6].

Dietary pattern research has identified strong evidence that diets high in vegetables, wholegrains, legumes, and other minimally processed foods are essential for reducing risk of CVD [5, 7]. Conversely, diets high in sugar-sweetened beverages, processed meat, or other ultra-processed foods have been associated with higher CVD risk [8]. Thus, the evidence for the benefits of minimally processed plant-based diets is growing, with many observational studies identifying their association with lower risk of CVD and its risk factors [9, 10]. Recent evidence from a meta-analysis of thirteen prospective cohort studies identified that healthy plant-based diets, i.e. those low in processed foods and beverages, were associated with a 13% lower risk of CVD incidence, but not mortality. Moreover, less healthy plant-based diets, which had greater consumption of processed foods, were associated with a 5% higher risk of CVD mortality, but no evidence of an association was found with CVD incidence [11]. Most previous research has adjusted for body mass index (BMI) as a confounder [11, 12], and found the relationships between plant-based diets and diabetes or ischemic heart disease were attenuated after adjustment for BMI [13, 14]. Given this, it is important to understand if fat mass may mediate the relationship between a plant-based diet and CVD, rather than confound it.

Understanding mediation in causal analysis is crucial for explaining how diets may improve CVD, as it outlines how the beneficial effects of healthy diets on CVD may be explained by mechanisms such as BMI or fat mass [15–18]. BMI has been shown to modify the relationship between diet and CVD [19, 20]. A previous umbrella review and meta-analysis found that high BMI is associated with increased CVD risk and acts as a causal risk factor [21]. However, few studies have examined specific measures of fat mass. Percentage body fat is an established risk factor for CVD and mortality [22, 23], and dual X-ray absorptiometry (DXA) provides an objective measure of this. Yet, evidence from DXA-based studies remains limited. A pooled analysis of seven prospective cohorts using bioelectrical impedance analysis (BIA) reported that fat mass was associated with a higher mortality risk. Importantly, this analysis highlighted that body composition, considering both fat and fat-free mass, predicted mortality better than BMI alone [24].

To date, no studies have examined whether the relationship between plant-based diet quality and incidence of CVD events and mortality is mediated by fat mass in mid-aged adults. This information is important to further understand the influence of key modifiable risk factors, which will guide evidence-based strategies to improve cardiometabolic health. Hence the aim of this study was to determine if longitudinal associations between plant-based diet quality indices and incidence of CVD events, CVD mortality, or all-cause mortality, were mediated by fat mass in mid-life.

## Methods

This study was reported according to the strengthening the reporting of observational studies in epidemiology - nutritional epidemiology (STROBE-nut) reporting guidelines [25], and recommended reporting criteria for mediation analysis with time-to-event outcomes [26] (Supplementary Tables1 and2).

### Study design and participants

Data from the UK Biobank, a prospective cohort study of mid-aged adults living in the UK was used, with details previously reported [27]. Nine million people were mailed using contact information from the National Health Service and were invited to participate in the study. This resulted in 500,000 people aged 40 to 69 years of age being recruited between 2006 and 2010 from across the UK. Participants attended an assessment centre at baseline between 2006 and 2010 where they signed electronic consent forms, completed questionnaires and interviews about diet and lifestyle behaviours, and had physical measures taken. The first participant follow-up was conducted in 2012–2013, second in 2014, and third in 2018. National health record data have been linked for deaths, cancers, hospital admissions and other disease-specific registers [27].

### Dietary intake

Dietary data were collected using the Oxford WebQ, a validated and self-administered dietary assessment tool that records the frequency of intake of over 200 foods and beverages consumed over the preceding 24 h [28]. It was first completed from April 2009 to September 2010. Following this, four online repeat cycles were run from February-April 2011 to April-June 2012. To calculate an estimate of usual intake, the four cycles from February 2011 to June 2012 were used [29]. Participants were excluded if they completed one dietary assessment as the Oxford WebQ has been validated for ≥2 assessments [30].

### Diet quality indices (exposure)

Diet quality was examined using three a priori plant-based diet quality indices, an overall plant-based diet (PDI), a healthy plant-based diet (hPDI), and a less healthy plant-based diet (uPDI) [18] (Supplementary Table 3). These indices were chosen as they are widely used for examining the relationship between plant-based diets and CVD [31]. Food and beverage items were allocated to the 17 index food groups. Population based quintiles of consumption (serves) of the 17 food groups were calculated using an average intake from the dietary assessments. Diet quality scores ranged from 17–85, and final scores were standardised to allow for comparison across the indices.

### CVD events and mortality (outcomes)

CVD mortality was determined using the International Classification of Diseases (I05-I89, 10th revision) [32]. CVD events included stroke and myocardial infarction, and were recorded at enrolment, up until the most recent inpatient hospital data (December 2022). CVD events, dates, and mortality dates were provided by the UK Biobank. Participants were excluded if they had a CVD event any time up to two years after their last dietary assessment to avoid potential for reverse causality [33]. At the time of the analysis, outcome data were available up until 17 December 2022. Hence, analysis was censored at this date, or the date of the outcome, whichever occurred first. Survival time for the outcomes were calculated from the last dietary assessment date until the censored date.

### Fat mass (mediator)

Percentage body fat measured by DXA was used as the measure for fat mass, using data from the first visit which commenced in 2014 [34]. Percentage body fat, derived from BIA was explored for the sensitivity analysis [35], with measurements from 2014 used.

### Covariates

Covariates considered as confounders were chosen based on previous literature [29, 36, 37], through analysis of the minimally adjusted model approach, and by using the directed acyclic graph (DAG) process (Supplementary Fig. 1). Sociodemographic confounders included age (continuous), sex (female/male), height (cm), Townsend Deprivation Index (continuous), and family history of cardiovascular diseases, diabetes or cancers (absence or presence of history from mother or father). Behavioural confounders comprised of alcohol intake (daily or almost daily, three or four times a week, once or twice a week or less), smoking status (current, previous, never or prefer not to answer) and physical activity measured as total metabolic equivalent of task (MET) hours per week (continuous). The analysis was adjusted for average energy intake, calculated as an individual average of the ≥2 dietary assessments (continuous). Covariates were tested for multicollinearity.

### Study sample

Participants were excluded if they (i) were pregnant, (ii) had <2 valid dietary assessments between 2011 and 2012 (within the range of 500–3500 kcal/d for women and 800–4200 kcal/d for men) [38], (ii) had a reported CVD event ≤2 years after their last dietary assessment, (iii) had missing data for exposures, outcomes, covariates, or mediators (Supplementary Fig. 2).

### Statistical analysis

Understanding sex-based differences in the context of this study is important as dietary intakes and the relationship between body composition and CVD differ between males and females [38–40]. Thus, due to differences in biology, risk factors, and previous evidence, the analysis was disaggregated per sex [41]. Characteristics of the total and final population were reported (Supplementary Table 4). Mean and standard error were reported for normally distributed data, and median and interquartile range (IQR) for skewed data. All plant-based diet quality indices were normally distributed. Dietary intake was assessed in 2011 to 2012, and DXA measurements were obtained in 2014, establishing temporal precedence of the exposure before the mediator and outcome, which strengthened the basis for causal inference. A negative control outcome analysis using accidental death to assess potential residual confounding was explored, however only one accidental death occurred during follow-up, precluding meaningful regression analysis.

**Fig. 1:**
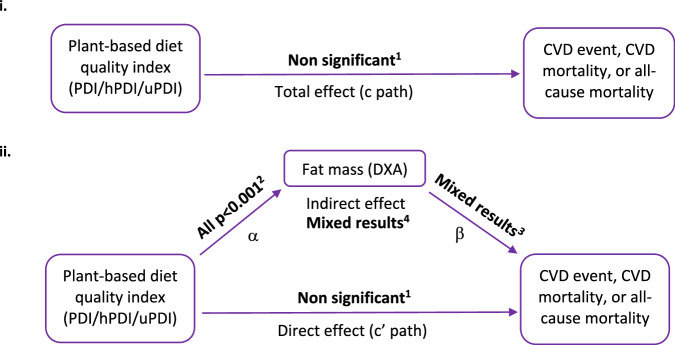
Mediation analysis models of association between adherence to a plant-based diet index and risk of CVD mortality, events, or all-cause mortality, mediated by fat mass. Path diagram i. outlines the total effect of the plant-based diet quality indices on CVD events, CVD mortality, or all-cause mortality, and diagram ii. outlines the indirect effect of the plant-based diet quality indices on CVD events, CVD mortality, or all-cause mortality through the fat mass mediator, and the direct effect of the plant-based diet quality indices on CVD events, CVD mortality, or all-cause mortality, after accounting for the fat mass mediator. ^1^ all p ≥ 0.1 ^2^ PDI and hPDI inversely associated with fat mass, uPDI positively associated with fat mass. ^3^ β paths with *p* < 0.05: PDI and CVD events (female only); uPDI and CVD events (all sexes); All diet quality indices (PDI/hPDI/uPDI) and CVD mortality (all sexes, and female only); hPDI and all-cause mortality (male only). ^4^ significant indirect effect paths: PDI/uPDI and CVD events via fat mass (female only); hPDI and CVD mortality via fat mass (all sexes); hPDI/uPDI and CVD mortality via fat mass (female only). All other b paths *p* ≥ 0.1.

To identify the association between the indices and all-cause mortality, CVD mortality, and CVD events, hazard ratios (HR) and 95% confidence intervals (CI) were calculated for each of the plant-based diet quality indices. Separate mediation analyses were used to identify the direct association between adherence to each of the plant-based diet quality indices and risk of CVD mortality, CVD events, or all-cause mortality, and the indirect effect mediated by fat mass [42]. Linear regression analyses were used to evaluate the relationship between the plant-based diet quality indices and fat mass (Path α, Fig. 1) (Supplementary Table 5). Cox proportional hazards models were used to assess the association between fat mass and CVD events, CVD mortality, or all-cause mortality (Path β, Fig. 1). A Cox model regression-based mediation analysis was used to identify the direct effect (plant-based diet quality indices on CVD mortality, CVD events, or all-cause mortality) and the indirect effect through fat mass. Indirect effects were estimated using the product of coefficients method. The observed coefficient for the indirect effect was calculated as the product of the regression coefficients for the α and β paths. Specifically, this was calculated by multiplying the coefficients for the exposure-mediator (linear regressions of each plant-based diet quality index on fat mass) and mediator-outcome (Cox regression of fat mass on CVD events, CVD mortality, or all-cause mortality) on the log scale, which was then exponentiated to a hazard ratio. This method was chosen as it provides valid estimates of indirect effects when the time-to-event outcome is rare ( < 10%) [26, 43]. Bootstrapping (1000 replications) was used to produce 95% confidence intervals (percentile method) for the indirect effect. For interpretability, the indirect effect per 2 standard deviation increase in the exposure was also reported. All Cox models estimating the direct effect (c′ path) included the mediator (fat mass), while models for the β path adjusted for the plant-based diet quality index exposure. Models for the total effect (c path) did not adjust for the mediator. Mediation analyses assume no unmeasured confounding of the exposure-outcome, exposure-mediator, and mediator-outcome relationships, correct model specification, and that the mediator is measured without error.

All models were tested for non-linear relationships. A sensitivity analysis was conducted using BIA-derived percentage body fat mass as the mediator. Analysis was performed using STATA (version 18). *P*-values assessed the strength of the evidence, with *p* < 0.001 indicating very strong evidence, *p* < 0.01 strong evidence, *p* < 0.05 moderate evidence, *p* < 0.1 weak evidence, and *p* ≥ 0.1 indicating insufficient evidence. As this was an exploratory analysis, no adjustments were made for multiplicity [44].

## Results

Of the 14,233 included participants (Supplementary Fig. 2), the mean age (at recruitment) was 55 years (SD ± 7.6), and 51.0% were female (Table 1). Almost three-quarters of participants (74.4%) had a family history of cardiovascular diseases, diabetes or cancers, and mean body fat was 32.7% (SD ± 8.2). New CVD events (*n* = 364, 2.6%), CVD mortality (*n* = 52, 0.4%) and all-cause mortality (*n* = 220, 1.6%) were identified during a mean follow-up of 11.6 (SD ± 0.4) years for CVD events, and 11.5 (SD ± 0.7) years for mortality. Mean plant-based diet quality scores were 50.5 (SD ± 5.9) for the PDI, 52.8 (SD ± 7.2) for the hPDI, and 54.0 (SD ± 6.8) for the uPDI, with females having slightly higher scores than males for the PDI and hPDI but not for the uPDI.

**Table 1:** Characteristics of participants in the UK Biobank included in this study (*n* = 14,233).

Characteristic	Overall	Females	Males
N (%)	14,233	7263 (51.0)	6970 (49.0)
Age at recruitment (years), mean ± SD	55.3 ( ± 7.6)	54.4 ( ± 7.4)	56.2 ( ± 7.7)
Height (cm), mean ± SD	170.3 ( ± 9.2)	163.8 ( ± 6.1)	177.0 ( ± 6.6)
Townsend deprivation index, median (IQR)	-2.6 (-3.9, -0.4)	-2.5 (-3.8, -0.3)	-2.7 (-4.0, -0.5)
Family history of cardiovascular diseases, diabetes, or cancers (n,%)			
Absence	3639 (25.6)	1845 (25.4)	1794 (25.7)
Presence	10,594 (74.4)	5418 (74.6)	5176 (74.3)
Alcohol intake (n,%)			
Daily or almost daily	3518 (24.7)	1472 (20.3)	2046 (29.4)
Three or four times a week	4033 (28.3)	1910 (26.3)	2123 (30.5)
Once or twice a week, or less	6682 (47.0)	3881 (53.4)	2801 (40.2)
Smoking status, n (%)			
Current	803 (5.6)	356 (4.9)	447 (6.4)
Previous	4726 (33.2)	2252 (31.0)	2474 (35.5)
Never or prefer not to answer	8704 (61.2)	4655 (64.1)	4049 (58.1)
Total MET hours, median (IQR)	28.3 (13.6, 51.6)	28.6 (13.8, 51.6)	27.8 (13.2, 51.7)
Fat mass (% body fat)^1^, mean ± SD	32.7 ( ± 8.2)	36.9 ( ± 7.4)	28.3 ( ± 6.5)
PDI, mean ± SD	50.5 ( ± 5.9)	50.9 ( ± 5.8)	50.2 ( ± 5.9)
hPDI, mean ± SD	52.8 ( ± 7.2)	54.2 ( ± 7.0)	51.3 ( ± 7.1)
uPDI, mean ± SD	54.0 ( ± 6.8)	53.7 ( ± 6.8)	54.2 ( ± 6.8)
Total energy intake (kj), median (IQR)	8681 (7395, 10165)	8068 (6959, 9328)	9410 (8073, 10960)
Number of new CVD events at follow-up, n (%)	364 (2.6)	109 (1.5)	255 (3.7)
Number of new CVD deaths at follow-up, n (%)	52 (0.4)	10 (0.1)	42 (0.6)
Number of new all-cause deaths at follow-up, n (%)	220 (1.6)	79 (1.1)	141 (2.0)

*PDI* plant-based diet index, *hPDI* healthy plant-based diet index, *uPDI* less healthy plant-based diet index, *SD* standard deviation, *IQR* interquartile range.^1%^ body fat by measured by DXA.

### CVD events

The path from the three plant-based diet quality indices to fat mass (Fig. 1, α path, and Supplementary Table 6) had strong evidence (*p* < 0.001) of an association in all models tested, with the PDI and hPDI inversely associated with fat mass, and the uPDI positively associated.

In the female only model, there was moderate evidence (*p* < 0.05) for an association between fat mass and CVD events, after controlling for the PDI (Fig. 1: β path, Supplementary Table 6). For the overall models (both males and females combined), there was moderate evidence (*p* < 0.05) for an association between fat mass and CVD events, after controlling for the uPDI.

#### Total, direct and indirect effects

There was no evidence of any total or direct effect paths for CVD events (Table 2). Although no overall association was observed between the plant-based diet indices and CVD events, mediation analysis was conducted to explore potential indirect pathways through fat mass. A negative indirect effect was found for the PDI on CVD events via fat mass for females (HR 0.978 (95% CI 0.958, 0.999)). A positive indirect effect was found for the uPDI on CVD events via fat mass for females (HR 1.029 (95% CI 1.003, 1.058)). The percentage mediated was not estimated due to a lack of total and direct effects.

**Table 2:** Mediation analysis of total, direct and indirect effects of plant-based diet quality indices and fat mass on CVD events, CVD mortality, or all-cause mortality (*n* = 14,233).

Mediator: fat mass (continuous)	Sex	*n* cases	Total effect (c path)		Direct effect (c’ path)		Indirect effect	
			(HR; 95% CI)*	*P*-value	(HR; 95% CI)*	*P*-value	(HR; 95% CI)	
							Per 1 SD*	Per 2 SD**
**CVD events**								
PDI	Overall	364	0.969 (0.871, 1.079)	0.567	0.980 (0.880, 1.091)	0.711	0.989 (0.976, 1.001)	0.977 (0.953, 1.002)
	Female	109	1.079 (0.881, 1.321)	0.464	1.098 (0.897, 1.344)	0.364	0.978 (0.958, 0.999)	0.957 (0.917, 0.999)
	Male	255	0.933 (0.822, 1.059)	0.284	0.940 (0.827, 1.067)	0.337	0.993 (0.977, 1.008)	0.987 (0.954, 1.017)
hPDI	Overall	364	0.979 (0.875, 1.095)	0.714	1.001 (0.893, 1.122)	0.992	0.977 (0.952, 1.001)	0.954 (0.906, 1.003)
	Female	109	0.951 (0.774, 1.167)	0.628	0.991 (0.804, 1.222)	0.933	0.956 (0.916, 1.003)	0.914 (0.839, 1.006)
	Male	255	0.996 (0.872, 1.138)	0.956	1.010 (0.882, 1.157)	0.886	0.986 (0.955, 1.014)	0.972 (0.911, 1.028)
uPDI	Overall	364	0.969 (0.870, 1.078)	0.560	0.955 (0.857, 1.064)	0.400	1.016 (1.000, 1.033)^a^	1.033 (1.000, 1.066)^a^
	Female	109	1.027 (0.843, 1.251)	0.790	0.997 (0.818, 1.216)	0.979	1.029 (1.003, 1.058)	1.059 (1.006, 1.118)
	Male	255	0.946 (0.832, 1.074)	0.390	0.937 (0.824, 1.065)	0.320	1.010 (0.991, 1.031)	1.021(0.983, 1.063)
**CVD mortality**								
PDI	Overall	52	1.032 (0.778, 1.370)	0.826	1.068 (0.804, 1.418)	0.651	0.962 (0.924, 1.001)	0.926 (0.853, 1.002)
	Female	10	0.807 (0.410, 1.589)	0.536	0.860 (0.442, 1.672)	0.656	0.911 (0.798, 1.002)	0.830 (0.636, 1.003)
	Male	42	1.087 (0.796, 1.485)	0.600	1.111 (0.812, 1.521)	0.509	0.977 (0.935, 1.023)	0.955 (0.873, 1.047)
hPDI	Overall	52	1.162 (0.863, 1.565)	0.323	1.251 (0.924, 1.694)	0.147	0.918 (0.846, 0.999)	0.843 (0.715, 0.996)
	Female	10	1.251 (0.636, 2.461)	0.516	1.478 (0.749, 2.919)	0.260	0.806 (0.603, 0.988)	0.650 (0.364, 0.976)
	Male	42	1.157 (0.829, 1.614)	0.391	1.210 (0.862, 1.698)	0.271	0.953 (0.876, 1.041)	0.909 (0.768, 1.083)
uPDI	Overall	52	0.966 (0.724, 1.288)	0.811	0.924 (0.691, 1.234)	0.592	1.052 (0.997, 1.111)	1.107 (0.994, 1.234)
	Female	10	0.945 (0.490, 1.824)	0.867	0.836 (0.432, 1.617)	0.594	1.139 (1.010, 1.355)	1.297 (1.019, 1.836)
	Male	42	0.969 (0.703, 1.336)	0.848	0.945 (0.685, 1.306)	0.733	1.028 (0.971, 1.088)	1.058 (0.942, 1.183)
**All-cause mortality**								
PDI	Overall	220	0.955 (0.831, 1.097)	0.515	0.964 (0.839, 1.108)	0.606	0.989 (0.971, 1.008)	0.978 (0.943, 1.015)
	Female	79	0.990 (0.780, 1.256)	0.933	0.992 (0.781, 1.260)	0.946	0.998 (0.966, 1.027)	0.995 (0.932, 1.055)
	Male	141	0.933 (0.786, 1.107)	0.425	0.947 (0.797, 1.125)	0.536	0.998 (0.966, 1.027)	0.995 (0.932, 1.055)
hPDI	Overall	220	1.052 (0.910, 1.215)	0.494	1.076 (0.929, 1.246)	0.328	0.975 (0.942, 1.012)	0.950 (0.887, 1.025)
	Female	79	0.989 (0.777, 1.259)	0.928	0.994 (0.777, 1.271)	0.959	0.995 (0.930, 1.062)	0.990 (0.865, 1.128)
	Male	141	1.091 (0.912, 1.307)	0.341	1.128 (0.940, 1.354)	0.195	0.995 (0.930, 1.062)	0.990 (0.865, 1.128)
uPDI	Overall	220	1.042 (0.907, 1.198)	0.558	1.028 (0.894, 1.183)	0.696	1.014 (0.989, 1.038)	1.029 (0.979, 1.077)
	Female	79	1.211 (0.960, 1.528)	0.106	1.212 (0.958, 1.533)	0.109	0.999 (0.960, 1.041)	0.999 (0.921, 1.085)
	Male	141	0.957 (0.805, 1.139)	0.622	0.938 (0.787, 1.117)	0.473	0.999 (0.960, 1.041)	0.999 (0.921, 1.085)

*HR* hazard ratios, *CI* confidence intervals, *PDI* plant-based diet index, *hPDI* healthy plant-based diet index, *uPDI* less healthy plant-based diet index. *Hazard Ratios with 95% Confidence Intervals (CI) for plant-based diet scores (1-standard deviation increments), ** Hazard Ratios with 95% Confidence Intervals (CI) for plant-based diet scores (2-standard deviation increments). Analysis adjusted for age at recruitment, height, Townsend deprivation index, alcohol intake, MET hours per week, family history of cardiovascular diseases, diabetes or cancers, smoking status, and average energy intake (additionally adjusted for sex in the non sex specific models). The total effect assessed the association between the PDI, hPDI and uPDI and all outcomes. Regression-based mediation analysis was used to identify the direct effect (plant-based diet quality indices on CVD mortality, CVD events, or all-cause mortality), and the indirect effect which was mediated by fat mass. Confidence intervals reported using rounded values for presentation. ^a^Statistical significance was evaluated using the unrounded estimates, which indicated that the indirect effect was not statistically significant, despite the rounded interval appearing to exclude the null value.

### CVD mortality

Across all models, all plant-based diet quality indices were associated with fat mass, with the PDI and hPDI inversely associated with fat mass, and the uPDI positively associated (Fig. 1, α path, and Supplementary Table 6).

In the overall (male and female combined) and female only models, there was moderate evidence (*p* < 0.05) for an association between fat mass and CVD mortality, after controlling for the plant-based diet quality indices separately (Fig. 1: β path, Supplementary Table 6).

#### Total, direct and indirect effects

For CVD mortality, there was no strong evidence of any total or direct effect paths (Table 2). In the overall model, there was a negative indirect effect for the hPDI on CVD mortality via fat mass (HR 0.918 (95% CI 0.846, 0.999)). In the female only models, a negative indirect effect was found for the hPDI (HR 0.806 (95% CI 0.603, 0.988)), and a positive indirect effect for the uPDI (HR 1.139 (95% CI 1.010, 1.355)).

### All-cause mortality

The three plant-based diet quality indices were associated with fat mass across all models, with the PDI and hPDI inversely associated with fat mass, and the uPDI positively associated (Fig. 1, α path, and Supplementary Table 6).

In the male only model, there was moderate evidence (*p* < 0.05) for an association between fat mass and all-cause mortality, after controlling for the hPDI (Fig. 1: β path, Supplementary Table 6).

#### Total, direct and indirect effects

There was no evidence of any total, direct, or indirect effect paths for all-cause mortality (Table 2).

### Sensitivity analysis

When BIA was used to derive the fat mass mediator, there were some differences in the indirect effect pathways (Supplementary Table 7). As per the main analysis, for the female only models there was a negative indirect effect of the PDI on CVD events, a positive indirect effect of the uPDI on CVD events (female only model), and a negative indirect effect of the hPDI on CVD mortality. In contrast to the main analysis, a significant negative indirect effect was found for the hPDI on CVD events for females only.

## Discussion

This study aimed to determine if longitudinal associations between plant-based diet quality indices and incidence of CVD events, CVD mortality, or all-cause mortality, were mediated by fat mass in mid-aged adults. There was no evidence for an association between the diet quality scores on the outcomes without, as well as after accounting for fat mass. However, it was found that the PDI and hPDI were inversely associated with fat mass, while the uPDI was positively associated. Greater fat mass was also associated with a higher risk of CVD events, CVD mortality, and all-cause mortality in several models after controlling for the plant-based diet quality indices. Additionally, fat mass demonstrated significant indirect effects on both CVD events and CVD mortality in several models. This indicates that higher plant-based diet quality scores may lower the risk of CVD events and mortality primarily by reducing fat mass, which is associated with an increased hazard of these outcomes when elevated. Hence, this study showed some evidence that fat mass may be a key mechanism linking plant-based diets to CVD.

There was no evidence of an overall association between the plant-based diet scores on CVD events, CVD mortality, or all-cause mortality. These results contrast to previous evidence. A recent systematic review and meta-analysis identified that higher plant-based diet quality was associated with a lower risk of CVD incidence and CVD mortality [45]. Additionally, previous UK Biobank studies found that a higher hPDI was associated with a reduced risk of cardiometabolic disorders [46], mortality, and CVD [37], regardless of genetic susceptibility [47]. Though, these studies had more participants ( > 100,000), and assessed diet quality using quantiles, which may explain the results discrepancy. However, in the present study, there was a significant negative indirect effect for the PDI or hPDI on CVD events or CVD mortality via the fat mass mediator, as well as a significant positive indirect effect for the uPDI. This evidence was found for the sexes combined and female only models and thus suggests that fat mass may play a role in explaining the relationship between plant-based diets with CVD events and CVD mortality. However, this also highlights that this relationship may be sex specific, as also evident in the sensitivity analysis.

This study found that all plant-based diet quality indices were related to fat mass, regardless of the healthiness of the diet. Previous studies have also observed a lower BMI for people following vegan/vegetarian diets [48, 49] compared to omnivorous diets, and meta-analyses have found following a vegetarian diet to be associated with reduced body weight [50, 51]. Despite this, reductions in weight do not necessarily result in reductions in fat, and evidence is lacking which uses comprehensive measures of body composition, such as DXA. A cohort study of mid-aged adults found a higher plant-based diet quality score was associated with lower fat mass over time (using DXA), regardless of the healthiness of the foods [52]. Additionally, cross-sectional data identified that adherence to the hPDI was associated with lower visceral adipose tissue, measured using magnetic resonance imaging [53], with no association found for the PDI or uPDI. Studies that derive plant-based diet quality indices and use high quality methods to assess fat mass are lacking, which limits a direct comparison with our findings.

This analysis identified some evidence that fat mass was related to a reduced risk of CVD events and CVD mortality, after controlling for the plant-based diet quality scores. An umbrella review and meta-analysis of observational and mendelian randomisation studies identified that a high fat mass was associated with increased CVD risk, and fat mass was also a causal risk factor for CVD [21]. However, BMI was used, rather than an objective measure. A pooled analysis of seven prospective cohorts assessed fat mass (using BIA), and identified that excess fat mass was associated with increased mortality risk [24]. Furthermore, the authors also identified that the relationship between BMI and mortality involves a combination of both individual effects from fat mass and fat-free mass, and that BMI showed less value for predicting mortality risk than did body composition. Hence, differences in results based on the measure of fat mass used is important to consider in public health and future research.

This study had several strengths. Dietary intake was assessed using three plant-based diet quality indices which enabled the healthiness of the diet to be assessed, not just the plant or animal origin. Additionally, national health record data was used, rather than self-report. The use of DXA and BIA, which are reliable measures of fat mass due to their direct and indirect methods, respectively, offered complementary insights into body composition. Moreover, the use of fat mass as a mediator has previously been highlighted as an important variable in mediation analysis, to ensure evidence is robust [26]. The analytical approaches for this study were also a strength. The mediator was assessed two years after the dietary exposure, and the longitudinal analysis included time varying variables which allowed for survival analysis to be undertaken.

There were several limitations to the present study. Baseline data on fat mass was only available in a small sample of participants and thus was not included. By not adjusting for this, it is unknown if fat mass changed, and in some cases, the follow-up time between the fat mass measure and the outcomes may have been minimal. However, given the mean follow-up of >11 years, this would have only impacted a small proportion of the sample. The dietary data in the UK Biobank should also be acknowledged as a potential limitation as the dietary assessments were self-reported and thus may reflect mis-reporting biases. Further to this, UK Biobank participants are generally healthier and more health-conscious than the general population, which may have introduced selection bias, and limits the generalisability of the study results. Additionally, the analysis for CVD mortality used a large number of covariates which given the sample size could result in unreliable results, with decreased statistical power. Nonetheless, this method was chosen using best practise DAG approaches and allowed for comparability across the analysis as a whole and modifying the covariates for each health outcome would limit the interpretation. It is also important to acknowledge that mediation analysis relies on key assumptions, including absence of unmeasured confounding and accurate measurement of the mediator; violations of these assumptions could bias the estimated indirect effects. Lastly, unmeasured and residual confounding from other sociodemographic, behaviour or dietary factors, such as smoking are also important to acknowledge.

The results from this study may have implications for public health. For example, recommendations to increase intake of healthy plant foods, and reduce intake of less healthy plant foods may be appropriate for those aiming for lower fat mass. Though, due to the lack of evidence found with health outcomes in the present study, and the evidence of weight stigma in this field, this should be advised with caution [54]. As this study found limited evidence of a mediating effect, future studies with longer follow-up time and larger sample size are required to determine whether there is a mediating effect from fat mass in the relationship between plant-based diets and CVD.

## Conclusion

This study found that the PDI and hPDI were inversely associated with fat mass, and fat mass was associated with survival in some models, after controlling for each of the indices. No evidence was found for an association between the plant-based diet quality indices on the outcomes without, as well as after accounting for fat mass. However, fat mass demonstrated significant indirect effects on both CVD events and CVD mortality in several models, suggesting that fat mass may be a key mechanism linking plant-based diets to CVD. Further studies are needed to understand the role of fat mass in the relationship between plant-based diets and CVD.

## Supplementary information

Supplementary Material

## Data Availability

Deidentified participant data may be obtained from the UK Biobank and is not publicly available. More information available at: https://www.ukbiobank.ac.uk/.
