# Supplementary material for: Plant-based diet quality, fat mass, and cardiovascular disease: A mediation analysis of mid-aged adults in the UK Biobank
Source: Eur J Clin Nutr. 2026 Apr 1;80(5):483–90. doi: 10.1038/s41430-026-01731-4 (PMC13186692; doi:10.1038/s41430-026-01731-4)
Supplement: Supplementary file 1 — Supplementary Material [file 41430_2026_1731_MOESM1_ESM.docx]

# Supplementary Material:

Supplementary material is intended for publication

### Supplementary Table 1: Strengthening the Reporting of Observational studies in Epidemiology – Nutritional Epidemiology (STROBE-nut) reporting guidelines (1)

| **Item** | **Item nr** | **STROBE recommendations** | **Extension for Nutritional Epidemiology studies (STROBE-nut)** | **Reported on page #** |
| --- | --- | --- | --- | --- |
| **Title and**  **abstract** | 1 | (a) Indicate the study’s design with a commonly used term in the title or the abstract.  (b) Provide in the abstract an informative and balanced summary of what was done and what was found. | **nut-1** State the dietary/nutritional assessment method(s) used in the title, abstract, or keywords. | 1-4  4-5 |
| **Introduction** |  |  |  | 5-7 |
| Background rationale | 2 | Explain the scientific background and rationale for the investigation being reported. |  | 5-7 |
| Objectives | 3 | State specific objectives, including any pre-specified hypotheses. |  | 7 |
| **Methods** |  |  |  | 8-12 |
| Study design | 4 | Present key elements of study design early in the paper. |  | 8 |
| Settings | 5 | Describe the setting, locations, and relevant dates, including periods of recruitment, exposure, follow-up, and data collection. | **nut-5** Describe any characteristics of the study settings that might affect the dietary intake or nutritional status of the participants, if applicable. | 8-9 |
| Participants | 6 | a) Cohort study—Give the eligibility criteria, and the sources and methods of selection of participants. Describe methods of follow-up.  Case-control study—Give the eligibility criteria, and the sources and methods of case ascertainment and control selection. Give the rationale for the choice of cases and controls.  Cross-sectional study—Give the eligibility criteria, and the sources and methods of selection of participants.  (b) Cohort study—For matched studies, give matching criteria and number of exposed and unexposed.  Case-control study—For matched studies, give matching criteria and the number of controls per case. | **nut-6** Report particular dietary, physiological or nutritional characteristics that were considered when selecting the target population. | 8-9  NA |
| Variables | 7 | Clearly define all outcomes, exposures, predictors, potential confounders, and effect modifiers. Give diagnostic criteria, if applicable. | **nut-7.1** Clearly define foods, food groups, nutrients, or other food components.  **nut-7.2** When using dietary patterns or indices, describe the methods to obtain them and their nutritional properties. | 9-11  9 |
| Data sources - measurements | 8 | For each variable of interest, give sources of data and details of methods of assessment (measurement).Describe comparability of assessment methods if there is more than one group. | **nut-8.1** Describe the dietary assessment method(s), e.g., portion size estimation, number of days and items recorded, how it was developed and administered, and how quality was assured. Report if and how supplement intake was assessed.  **nut-8.2** Describe and justify food composition data used. Explain the procedure to match food composition with consumption data. Describe the use of conversion factors, if applicable.  **nut-8.3** Describe the nutrient requirements, recommendations, or dietary guidelines and the evaluation approach used to compare intake with the dietary reference values, if applicable.  **nut-8.4** When using nutritional biomarkers, additionally use the STROBE Extension for Molecular Epidemiology (STROBE-ME). Report the type of biomarkers used and their usefulness as dietary exposure markers.  **nut-8.5** Describe the assessment of nondietary data (e.g., nutritional status and influencing factors) and timing of the assessment of these variables in relation to dietary assessment.  **nut-8.6** Report on the validity of the dietary or nutritional assessment methods and any internal or external validation used in the study, if applicable. | 8-9  NA  NA  NA  NA  8-9 |
| Bias | 9 | Describe any efforts to address potential sources of bias. | **nut-9** Report how bias in dietary or nutritional assessment was addressed, e.g., misreporting, changes in habits as a result of being measured, or data imputation from other sources | 8-11 |
| Study Size | 10 | Explain how the study size was arrived at. |  | 11 |
| Quantitative variables | 11 | Explain how quantitative variables were handled in the analyses. If applicable, describe which groupings were chosen and why. | **nut-11** Explain categorization of dietary/nutritional data (e.g., use of N-tiles and handling of nonconsumers) and the choice of reference category, if applicable. | 9 |
| Statistical  Methods | 12 | (a) Describe all statistical methods, including those used to control for confounding  (b) Describe any methods used to examine subgroups and interactions.  (c) Explain how missing data were addressed.  (d) Cohort study—If applicable, explain how loss to follow-up was addressed.  Case-control study—If applicable, explain how matching of cases and controls was addressed.  Cross-sectional study—If applicable, describe analytical methods taking account of sampling strategy.  (e) Describe any sensitivity analyses. | **nut-12.1** Describe any statistical method used to combine dietary or nutritional data, if applicable.  **nut-12.2** Describe and justify the method for energy adjustments, intake modeling, and use of weighting factors, if applicable.  **nut-12.3** Report any adjustments for measurement error, i.e, from a validity or calibration study. | NA  11  11  14 |
| **Results** |  |  |  | 13-16 |
| Participants | 13 | (a) Report the numbers of individuals at each stage of the study—e.g., numbers potentially eligible, examined for eligibility, confirmed eligible, included in the study, completing follow-up, and analyzed.  (b) Give reasons for non-participation at each stage.  (c) Consider use of a flow diagram. | **nut-13** Report the number of individuals excluded based on missing, incomplete or implausible dietary/nutritional data. | Supplementary Figure 2 |
| Descriptive data | 14 | (a) Give characteristics of study participants (e.g., demographic, clinical, social) and information on exposures and potential confounders  (b) Indicate the number of participants with missing data for each variable of interest  (c) Cohort study—Summarize follow-up time (e.g., average and total amount) | **nut-14** Give the distribution of participant characteristics across the exposure variables if applicable. Specify if food consumption of total population or consumers only were used to obtain results. | Table 1 |
| Outcome data | 15 | Cohort study—Report numbers of outcome events or summary measures over time.  Case-control study—Report numbers in each exposure category, or summary measures of exposure.  Cross-sectional study—Report numbers of outcome events or summary measures. |  | Table 1 |
| Main results | 16 | (a) Give unadjusted estimates and, if applicable, confounder-adjusted estimates and their precision (e.g., 95% confidence interval).  Make clear which confounders were adjusted for and why they were included.  (b) Report category boundaries when continuous variables were categorized.  (c) If relevant, consider translating estimates of relative risk into absolute risk for a meaningful time period. | **nut-16** Specify if nutrient intakes are reported with or without inclusion of dietary supplement intake, if applicable. | NA |
| Other analyses | 17 | Report other analyses done—e.g., analyses of subgroups and interactions and sensitivity analyses. | **nut-17** Report any sensitivity analysis (e.g., exclusion of misreporters or outliers) and data imputation, if applicable. | 15-16 |
| **Discussion** |  |  |  | 17-22 |
| Key results | 18 | Summarize key results with reference to study objectives. |  | 17 |
| Limitation | 19 | Discuss limitations of the study, taking into account sources of potential bias or imprecision. Discuss both direction and magnitude of any potential bias. | **nut-19** Describe the main limitations of the data sources and assessment methods used and implications for the interpretation of the findings. | 21-22 |
| Interpretation | 20 | Give a cautious overall interpretation of results considering objectives, limitations, multiplicity of analyses, results from similar studies, and other relevant evidence. | **nut-20** Report the nutritional relevance of the findings, given the complexity of diet or nutrition as an exposure. | 17-22 |
| Generalizability | 21 | Discuss the generalizability (external validity) of the study results. |  |  |
| **Other information** |  |  |  |  |
| Funding | 22 | Give the source of funding and the role of the funders for the present study and, if applicable, for the original study on which the present article is based. |  | 2 |
| *Ethics* |  |  | **nut-22.1** Describe the procedure for consent and study approval from ethics committee(s). | 30 |
| *Supplementary material* |  |  | **nut-22.2** Provide data collection tools and data as online material or explain how they can be accessed. | Separate document |

### Supplementary Table 2: Recommended reporting criteria for mediation analysis with time-to-event outcomes (2)

| Section | Recommendation | Location in paper |
| --- | --- | --- |
| Objectives | State whether mediation analysis(es) is/are exploratory or hypothesis-based | Introduction |
| Methods | Specify criteria or statistical tests used to assess mediation, with references  *Was the goal to categorize mediation as absent, partial or complete, or to estimate exact values for direct and indirect effects*? | Methods - Statistical analysis |
|  | Detail how exposure, mediator and outcome variables were defined and measured | Methods |
|  | Detail when exposure, mediator and outcome variables were measured | Methods |
|  | Describe statistical models used for the mediator(s) and outcome(s), and any assumptions underlying use of such models (e.g. proportionality, rare outcome assumption for Cox Proportional Hazards models) | Methods - Statistical analysis |
|  | State whether interaction between exposure and mediator was considered, and how | Methods - Statistical analysis |
|  | Reference any software programs used for mediation analysis | Methods - Statistical analysis |
|  | If relevant for exposure, mediator, and outcome being considered, state how the following were addressed:  - clustering or repeated events  - competing risks | NA |
|  | Describe assumptions underlying mediation analysis, and methods used to address these (e.g.: randomisation, regression, weighting, stratification, sensitivity analysis) | Methods - Statistical analysis |
| Results | Report measures of mediation effect (indirect effect or proportion mediated) accompanied by 95% confidence intervals | Table 2 |
|  | Report p-values for mediation hypothesis testing | Table 2 and Figure 1 |
| Discussion | Discuss limitations of causal inference based on mediation analysis results, including whether underlying assumptions were met  Discuss magnitude and direction of any potential bias | Discussion |

### Supplementary Table 3: Food items from the UK Biobank OxfordWebQ assigned to the 17 food groups, and their scoring across the three plant-based diet quality indices

|  | | **PDI** | **hPDI** | **uPDI** |
| --- | --- | --- | --- | --- |
| **Plant Food Groups** | | | | |
| **Healthy** | | | | |
| Whole grains | Porridge, muesli, bran cereal, whole-wheat cereal, wholemeal pasta, brown rice, sliced bread*, baguette*, bap*, bread roll* | Positive scores | Positive scores | Reverse scores |
| Fruits | Stewed fruit, prune, dried fruit, mixed fruit, apple, banana, berry, cherry, grapefruit, grape, mango, melon, orange, satsuma, peach/nectarine, pear, pineapple, plum, other fruit | Positive scores | Positive scores | Reverse scores |
| Vegetables | Avocado, beetroot, broad bean, broccoli, butternut squash, cabbage/kale, carrot, cauliflower, celery, coleslaw, courgette, cucumber, fresh tomato, garlic, green bean, leek, lettuce, mixed vegetable, mushroom, olives, onion, other vegetables, parsnip, pea, side salad, spinach, sprouts, sweet pepper, sweet potato, sweetcorn, tinned tomato, turnip/swede, vegetable pieces, watercress, canned or homemade soup* | Positive scores | Positive scores | Reverse scores |
| Nuts | Seeds, salted peanuts, unsalted peanuts, salted nuts, unsalted nuts | Positive scores | Positive scores | Reverse scores |
| Legumes | Vegetarian sausages/burgers, tofu, Quorn, other vegetarian alternatives, baked beans, pulses, plant-based milk*, canned or homemade soup*, plant-based milk added to cereal/tea/coffee* | Positive scores | Positive scores | Reverse scores |
| Tea and coffee | Cappuccino, espresso, filtered coffee, green tea, herbal tea, instant coffee, latte, other coffee type, other tea, rooibos tea, standard tea | Positive scores | Positive scores | Reverse scores |
| **Less healthy** | | | | |
| Fruit juices | Orange juice, grapefruit juice, pure fruit/vegetable juice, fruit smoothie | Positive scores | Reverse scores | Positive scores |
| Refined grains | Other grain, oat crunch, plain cereal, other cereal, crispbread, other bread, couscous, sweetened cereal, naan bread, garlic bread, croissant, scone, other savoury snack, pancake, scotch pancake, yorkshire pudding, savoury biscuits, cheesy biscuits, white pasta, white rice, snackpot, oatcakes, cereal bar, canned or homemade soup*, sliced bread*, baguette*, bap*, bread roll* | Positive scores | Reverse scores | Positive scores |
| Potatoes | Crisp, fried potatoes, boiled/baked potatoes, mashed potato | Positive scores | Reverse scores | Positive scores |
| Sugar sweetened beverages | Squash, low calorie drink, fizzy drink | Positive scores | Reverse scores | Positive scores |
| Sweets and desserts | Danish pastry, double crust pastry, single crust pastry, crumble, milk-based pudding, other milk-based pudding, soya dessert, fruitcake, cake, doughnut, sponge pudding, cheesecake, other dessert, chocolate bar, white chocolate, milk chocolate, dark chocolate, chocolate-covered raisin, chocolate sweet, diet sweets, sweets, chocolate-covered biscuits, chocolate biscuits, sweet biscuits, other sweets | Positive scores | Reverse scores | Positive scores |
| **Animal Food Groups** | | | | |
| Animal fat | Number of baguettes/baps/bread rolls/bread slices/crackers/crispbreads/oatcakes/other bread types with butter/margarine, and thickness of butter/margarine spread on baguettes/bread rolls/crackers/crispbreads/large baps/oatcakes/ other bread/sliced bread | Reverse scores | Reverse scores | Reverse scores |
| Dairy | Blue cheese, cheese spread, cottage cheese, dairy smoothie, feta, flavored milk, goat's cheese, hard cheese, hot chocolate, ice-cream, low calorie hot chocolate, low fat cheese spread, low fat hard cheese, animal sourced milk*, animal sourced milk added to cereal/tea/coffee*, mozzarella, other cheese, soft cheese, yogurt | Reverse scores | Reverse scores | Reverse scores |
| Egg | Whole egg, omelette, eggs in sandwiches, other egg, scotch egg | Reverse scores | Reverse scores | Reverse scores |
| Fish or seafood | Tinned tuna, oily fish, breaded fish, battered fish, white fish, prawns, lobster/crab, shellfish, other fish, canned or homemade soup* | Reverse scores | Reverse scores | Reverse scores |
| Meat | Sausage, beef, pork, lamb, crumbed or deep-fried poultry, poultry, bacon, ham, liver, other meat, canned or homemade soup* | Reverse scores | Reverse scores | Reverse scores |
| Miscellaneous animal-based foods | Indian snacks, sushi, pizza | Reverse scores | Reverse scores | Reverse scores |

PDI, plant-based diet index; hPDI, healthy plant-based diet index; uPDI, less healthy plant-based diet index. To classify grains as either refined or whole grains, foods were assumed to be refined grains unless they were specified as brown or wholegrain. Milk items were disaggregated into plant-based or animal-sourced varieties, and breads were disaggregated into refined or wholegrain categories. Soups were categorised by their main ingredient of pulses, meat, vegetables, fish or seafood, and/or pasta. Food items such as sauces and condiments, added sugars, and added salt were not included as they were not captured in the indices. If the food items were not provided in a serve quantity, a serve was calculated using standard measurements data (3). As per previous studies, (4-9) the vegetable oil food group was excluded as this was not collected in the Oxford WebQ. *disaggregated item.

### Supplementary Figure 1: Directed acyclic graph (DAG) used to help identify the covariate selection for the statistical analysis


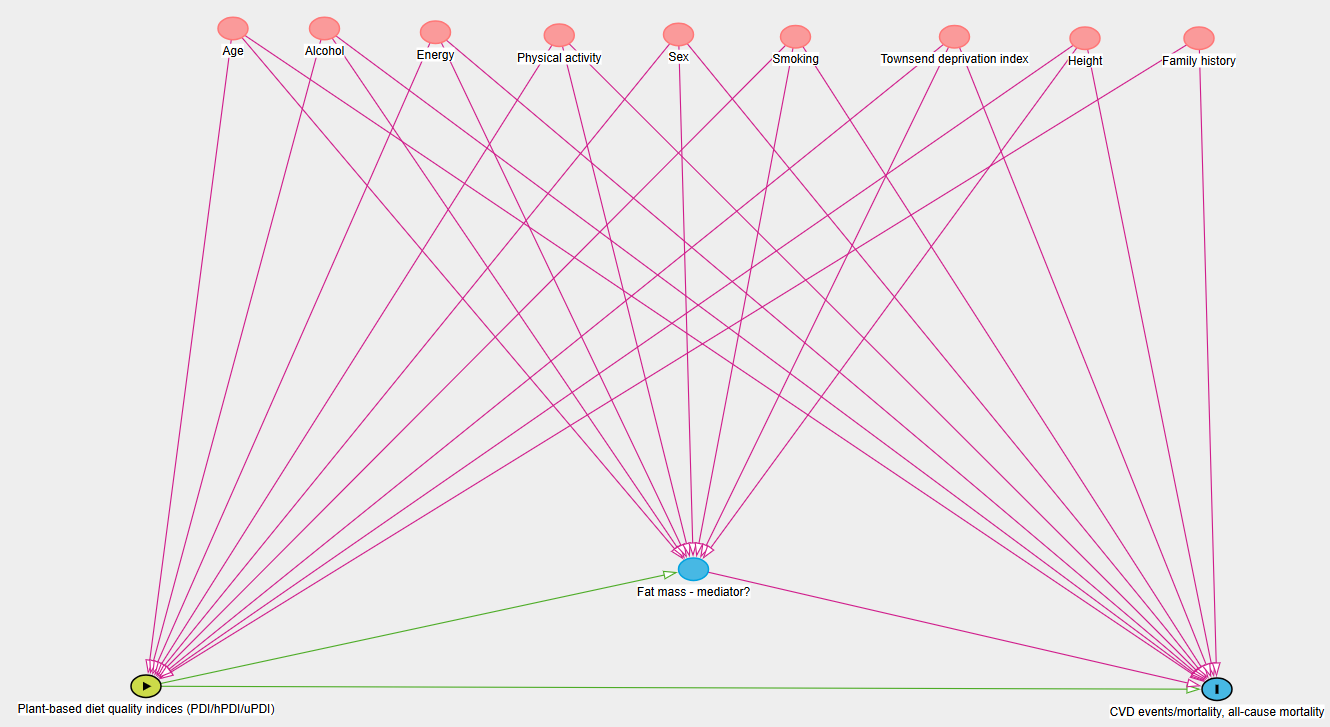


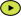
  exposure
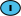
  outcome
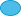
  ancestor of outcome
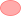
  ancestor of exposure *and* outcome
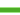
  causal path
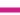
  biasing path

### Supplementary Figure 2: Diagram of participants included in the mediation analysis

Consenting participants in the UK Biobank (n=502,357)

Excluded (n=23,272)

- Completed less than 2 days of the

OxfordWebQ (n=23,272)

Participants included in the analysis (n=14,233)

Completed at least 2 dietary assessments (n=17,019)

Mediator data available

(n=40,291)

Not included (n=462,066)

- No data for mediator (fat mass) (n=462,066)

Excluded (did not meet inclusion criteria)* (n=518)

- Pregnancy (n=12)
- CVD event prior, or up until two years after last dietary assessment(n=376)
- Mortality up until two years after last dietary assessment (n=0)
- <2 valid OxfordWebQ (n=133)

Excluded (missing data)* (n=2,346)

- Townsend deprivation index (n=17)
- Total MET hours (n=2,318)
- Height (n=16)
- Smoking status (n=7)
- Alcohol intake (n=0)
- Family history (n=0)

*categories are not mutually exclusive

### Supplementary Table 4: Characteristics of participants in the UK Biobank included and excluded in this study

| **Characteristic** | **Included 14,233 (2.8%)** | **Excluded 488,124 (97.2%)** |
| --- | --- | --- |
| Age at recruitment (years), mean ±SD | 55.3 (7.6) | 56.6 (±8.1) |
| Female | 7,263 (51.0) | 266,031 (54.5) |
| Highest level of education ^a^ |  |  |
| College or university degree | 7,815 (54.9) | 153,285 (31.7) |
| A levels/AS levels or equivalent | 1,929 (13.6) | 53,372 (11.0) |
| O levels/GCSE/CSEs or equivalent | 2,725 (19.2) | 129,326 (26.8) |
| Professional qualifications (NVQ/HND/HNC, other professional qualifications) | 1,220 (8.6) | 57,299 (11.9) |
| None/prefer not to answer | 544 (3.8) | 90,200 (18.7) |
| Townsend deprivation index^b^ | -1.8 (2.8) | -1.3 (3.1) |
| Smoking^c^ |  |  |
| Previous | 4,726 (33.2) | 168,276 (34.54) |
| Current | 803 (5.6) | 52,155 (10.70) |
| Never or prefer not to answer | 8,704 (61.2) | 266,800 (54.8) |
| Ethnicity |  |  |
| British, Irish, or any other white background | 13,778 (96.8) | 458,784 (94.0) |
| All other ethnicities | 455 (3.2) | 29,340 (6.0) |
| Family history of cardiovascular diseases, diabetes or cancers |  |  |
| Absence | 3,639 (25.6) | 129,582 (26.6) |
| Presence | 10,594 (74.4) | 358,542 (73.5) |
| Alcohol intake |  |  |
| Daily or almost daily | 3,518 (24.7) | 98,226 (20.1) |
| Three or four times a week | 4,033 (28.3) | 111,379 (22.8) |
| Once or twice a week, or less | 6,682 (47.0) | 278,519 (57.1) |
| Total MET hours, mean ±SD^d^ | 39.1 (37.3) | 44.3 (±44.4) |
| % body fat, mean ±SD^e^ | 32.7 (8.2) | 34.0 (±8.1) |
| Total energy intake, mean ±SD^f^ | 8902 (2173) | 8851 (±2679) |

All values are n (%) unless otherwise specified. Excluded categories comprised of: ^a^ n=483,482, ^b^ n= 487,498, ^c^ n= 487,231, ^d^ n=370,944, ^e^ n=26,058, ^f^ n=161,687

### Supplementary Table 5: Mediation analysis methods

| Mediation analysis path (Figure 1) | Methodology used |
| --- | --- |
| Path α | Linear regression analyses were used to evaluate the relationship between the plant-based diet quality indices and fat mass. |
| Path β | Cox proportional hazards models were used to assess the association between fat mass and CVD events, CVD mortality, or all-cause mortality |
| Direct effect | A Cox model regression-based mediation analysis was used to identify the direct effect (plant-based diet quality indices on CVD mortality, CVD events, or all-cause mortality). |
| Indirect effect | Indirect effects were estimated as the product of coefficients from linear regressions of each plant-based diet index on fat mass and the Cox regression of fat mass on CVD events, CVD mortality, or all-cause mortality. This method was chosen as it provides valid estimates of indirect effects when the time-to-event outcome is rare (<10%) (2, 10). Bootstrapping (1000 replications) was used to produce 95% confidence intervals (percentile method) for the indirect effect. The observed coefficient for the indirect effect was calculated as the product of the regression coefficients for the α and β paths and exponentiated to a hazard ratio. |

### Supplementary Table 6: Mediation analysis α and β path results of the association between adherence to a plant-based diet index and risk of CVD mortality, events, or all-cause mortality, mediated by fat mass (n=14,233)

|  | Sex | n events | α path | | β path | |
| --- | --- | --- | --- | --- | --- | --- |
|  |  |  | Coefficient; 95% CI | P-value | HR; 95% CI | P-value |
| CVD events | | | | | | |
| PDI | Overall | 364 | -0.739 (-0.855, -0.624) | **0.000** | 1.016 (1.000, 1.032) | 0.056 |
|  | Female | 109 | -0.778 (-0.952, -0.604) | **0.000** | 1.029 (1.001, 1.058) | **0.042** |
|  | Male | 255 | -0.714 (-0.866, -0.561) | **0.000** | 1.009 (1.000, 1.030) | 0.355 |
| hPDI | Overall | 364 | -1.488 (-1.606, -1.370) | **0.000** | 1.016 (1.000, 1.032) | 0.055 |
|  | Female | 109 | -1.648 (-1.824, -1.477) | **0.000** | 1.028 (0.999, 1.057) | 0.056 |
|  | Male | 255 | -1.338 (-1.494, -1.182) | **0.000** | 1.011 (0.991, 1.031) | 0.295 |
| uPDI | Overall | 364 | 0.963 (0.849, 1.078) | **0.000** | 1.017 (1.001, 1.033) | **0.041** |
|  | Female | 109 | 1.039 (0.870, 1.209) | **0.000** | 1.028 (1.000, 1.057) | 0.051 |
|  | Male | 255 | 0.886 (0.734, 1.038) | **0.000** | 1.012 (0.992, 1.032) | 0.249 |
| CVD mortality | | | | | | |
| PDI | Overall | 52 | -0.739 (-0.855, -0.624) | **0.000** | 1.054 (1.009, 1.101) | **0.019** |
|  | Female | 10 | -0.778 (-0.952, -0.604) | **0.000** | 1.127 (1.022, 1.244) | **0.017** |
|  | Male | 42 | -0.714 (-0.866, -0.561) | **0.000** | 1.033 (0.983, 1.086) | 0.204 |
| hPDI | Overall | 52 | -1.488 (-1.606, -1.370) | **0.000** | 1.059 (1.013, 1.107) | **0.011** |
|  | Female | 10 | -1.648 (-1.824, -1.473) | **0.000** | 1.139 (1.031, 1.259) | **0.010** |
|  | Male | 42 | -1.338 (-1.494, -1.182) | **0.000** | 1.036 (0.985, 1.090) | 0.165 |
| uPDI | Overall | 52 | 0.963 (0.849, 1.078) | **0.000** | 1.054 (1.009, 1.101) | **0.018** |
|  | Female | 10 | 1.039 (0.870, 1.209) | **0.000** | 1.133 (1.026, 1.252) | **0.014** |
|  | Male | 42 | 0.886 (0.734, 1.038) | **0.000** | 1.032 (0.982, 1.085) | 0.215 |
| All-cause mortality | | | | | | |
| PDI | Overall | 220 | -0.739 (-0.855, -0.624) | **0.000** | 1.015 (0.994, 1.036) | 0.153 |
|  | Female | 79 | -0.778 (-0.952, -0.604) | **0.000** | 1.003 (0.972, 1.036) | 0.845 |
|  | Male | 141 | -0.714 (-0.866, -0.561) | **0.000** | 1.024 (0.997, 1.051) | 0.086 |
| hPDI | Overall | 220 | -1.488 (-1.606, -1.370) | **0.000** | 1.017 (0.997, 1.039) | 0.102 |
|  | Female | 79 | -1.648 (-1.824, -1.473) | **0.000** | 1.003 (0.971, 1.036) | 0.852 |
|  | Male | 141 | -1.338 (-1.494, -1.182) | **0.000** | 1.028 (1.000, 1.056) | **0.046** |
| uPDI | Overall | 220 | 0.963 (0.849, 1.078) | **0.000** | 1.015 (0.994, 1.036) | 0.156 |
|  | Female | 79 | 1.039 (0.870, 1.209) | **0.000** | 0.999 (0.968, 1.032) | 0.970 |
|  | Male | 141 | 0.886 (0.734, 1.038) | **0.000** | 1.026 (0.999, 1.054) | 0.062 |

Abbreviations: HR, hazard ratios; CI, confidence intervals; PDI, plant-based diet index; hPDI, healthy plant-based diet index; uPDI, less healthy plant-based diet index.

### Supplementary Table 7: Sensitivity mediation analysis between plant-based diet quality indices and fat mass measured using BIA, on CVD events, CVD mortality, or all-cause mortality (n=19,058)

| Mediator: fat mass (continuous) | Sex | n events | **Total effect (c path)** | | **Direct effect (c’ path)** | | **Indirect effect** | |
| --- | --- | --- | --- | --- | --- | --- | --- | --- |
|  |  |  | (HR; 95% CI)*  (c path) | P-value | (HR; 95% CI)*  (c’ path) | P-value | (HR; 95% CI) | |
|  |  |  |  |  |  |  | Per 1 SD* | Per 2 SD** |
| CVD events | | | | | | | | |
| PDI | Overall | 483 | 0.939 (0.856, 1.031) | 0.187 | 0.948 (0.864, 1.041) | 0.266 | 0.990 (0.979, 1.000) | 0.980 (0.956, 1.002) |
|  | Female | 151 | 1.058 (0.893, 1.254) | 0.514 | 1.077 (0.908, 1.277) | 0.394 | 0.979 (0.961, 0.995) | 0.958 (0.923, 0.990) |
|  | Male | 332 | 0.897 (0.803, 1.003) | 0.056 | 0.900 (0.804, 1.007) | 0.065 | 0.997 (0.983, 1.013) | 0.994 (0.966, 1.026) |
| hPDI | Overall | 483 | 0.938 (0.850, 1.034) | 0.198 | 0.953 (0.863, 1.053) | 0.347 | 0.982 (0.962, 1.003) | 0.965 (0.925, 1.005) |
|  | Female | 151 | 0.925 (0.778, 1.010) | 0.377 | 0.961 (0.806, 1.147) | 0.663 | 0.960 (0.925, 0.994) | 0.922 (0.856, 0.987) |
|  | Male | 332 | 0.946 (0.840, 1.065) | 0.357 | 0.951 (0.843, 1.073) | 0.414 | 0.994 (0.972, 1.020) | 0.989 (0.945, 1.041) |
| uPDI | Overall | 483 | 1.000 (0.911, 1.097) | 0.994 | 0.988 (0.900, 1.085) | 0.807 | 1.012 (1.000, 1.025) ^a^ | 1.025 (0.999, 1.050) |
|  | Female | 151 | 1.076 (0.910, 1.271) | 0.393 | 1.048 (0.886, 1.239) | 0.586 | 1.024 (1.005, 1.047) | 1.050 (1.009, 1.097) |
|  | Male | 332 | 0.970 (0.867, 1.084) | 0.585 | 0.965 (0.862, 1.080) | 0.531 | 1.005 (0.989, 1.020) | 1.010 (0.978, 1.041) |
| CVD mortality | | | | | | | | |
| PDI | Overall | 59 | 1.017 (0.779, 1.327) | 0.902 | 1.037 (0.794, 1.356) | 0.789 | 0.978 (0.941, 1.017) | 0.957 (0.886, 1.034) |
|  | Female | 12 | 0.817 (0.447, 1.492) | 0.510 | 0.865 (0.475, 1.575) | 0.634 | 0.931 (0.846, 1.001) | 0.867 (0.715, 1.002) |
|  | Male | 47 | 1.071 (0.796, 1.441) | 0.650 | 1.075 (0.798, 1.449) | 0.634 | 0.996 (0.953, 1.042) | 0.992 (0.907, 1.087) |
| hPDI | Overall | 59 | 1.136 (0.857, 1.506) | 0.375 | 1.181 (0.887, 1.573) | 0.255 | 0.955 (0.885, 1.031) | 0.911 (0.783, 1.062) |
|  | Female | 12 | 1.003 (0.542, 1.855) | 0.993 | 1.146 (0.612, 2.147) | 0.670 | 0.857 (0.704, 0.992) | 0.735 (0.496, 0.984) |
|  | Male | 47 | 1.179 (0.858, 1.620) | 0.310 | 1.190 (0.862, 1.643) | 0.290 | 0.989 (0.919, 1.072) | 0.979 (0.844, 1.149) |
| uPDI | Overall | 59 | 1.018 (0.776, 1.334) | 0.900 | 0.997 (0.759, 1.309) | 0.983 | 1.025 (0.977, 1.072) | 1.051 (0.955, 1.149) |
|  | Female | 12 | 1.260 (0.692, 2.294) | 0.449 | 1.158 (0.641, 2.092) | 0.628 | 1.090 (1.000, 1.221) ^a^ | 1.187 (1.000, 1.490) ^a^ |
|  | Male | 47 | 0.964 (0.711, 1.306) | 0.812 | 0.961 (0.708, 1.305) | 0.798 | 1.004 (0.956, 1.053) | 1.007 (0.914, 1.108) |
| All-cause mortality | | | | | | | | |
| PDI | Overall | 279 | 0.931 (0.823, 1.053) | 0.255 | 0.935 (0.826, 1.058) | 0.286 | 0.995 (0.978, 1.011) | 0.990 (0.957, 1.022) |
|  | Female | 96 | 0.937 (0.756, 1.161) | 0.551 | 0.937 (0.756, 1.161) | 0.551 | 1.000 (0.975, 1.028) | 1.000 (0.952, 1.057) |
|  | Male | 183 | 0.924 (0.795, 1.074) | 0.302 | 0.929 (0.798, 1.081) | 0.340 | 1.000 (0.975, 1.028) | 1.000 (0.952, 1.057) |
| hPDI | Overall | 279 | 1.063 (0.935, 1.209) | 0.347 | 1.076 (0.944, 1.225) | 0.274 | 0.987 (0.955, 1.017) | 0.974 (0.912, 1.035) |
|  | Female | 96 | 1.028 (0.826, 1.278) | 0.807 | 1.001 (0.970, 1.034) | 0.797 | 0.998 (0.947, 1.055) | 0.996 (0.897, 1.114) |
|  | Male | 183 | 1.086 (0.927, 1.273) | 0.307 | 1.100 (0.937, 1.292) | 0.245 | 0.998 (0.947, 1.055) | 0.996 (0.897, 1.114) |
| uPDI | Overall | 279 | 1.004 (0.887, 1.135) | 0.955 | 0.998 (0.881, 1.129) | 0.970 | 1.006 (0.987, 1.026) | 1.013 (0.975, 1.053) |
|  | Female | 96 | 1.125 (0.912, 1.388) | 0.271 | 1.127 (0.912, 1.394) | 0.269 | 0.998 (0.967, 1.030) | 0.997 (0.935, 1.061) |
|  | Male | 183 | 0.942 (0.809, 1.097) | 0.443 | 0.935 (0.803, 1.090) | 0.392 | 0.998 (0.967, 1.030) | 0.997 (0.935, 1.061) |

Abbreviations: HR, hazard ratios; CI, confidence intervals; PDI, plant-based diet index; hPDI, healthy plant-based diet index; uPDI, less healthy plant-based diet index. *Hazard Ratios with 95% Confidence Intervals (CI) for plant-based diet scores (1-standard deviation increments), ** Hazard Ratios with 95 % Confidence Intervals (CI) for plant-based diet scores (2-standard deviation increments). Analysis adjusted for age at recruitment, Townsend deprivation index, alcohol intake, MET hours per week, family history of cardiovascular diseases, diabetes or cancers, and average energy intake (additionally adjusted for sex in the non sex specific models). The total effect assessed the association between the PDI, hPDI and uPDI and all outcomes. Regression-based mediation analysis was used to identify the direct effect (plant-based diet quality indices on CVD mortality, CVD events, or all-cause mortality), and the indirect effect which was mediated by fat mass. Confidence intervals reported using rounded values for presentation. ^a^ Statistical significance was evaluated using the unrounded estimates, which indicated that the indirect effect was not statistically significant, despite the rounded interval appearing to exclude the null value.

**References:**

1. Lachat C, Hawwash D, Ocke MC, Berg C, Forsum E, Hornell A, et al. Strengthening the Reporting of Observational Studies in Epidemiology - nutritional epidemiology (STROBE-nut): An extension of the STROBE statement. Nutr Bull. 2016;41(3):240-51.

2. Lapointe-Shaw L, Bouck Z, Howell NA, Lange T, Orchanian-Cheff A, Austin PC, et al. Mediation analysis with a time-to-event outcome: a review of use and reporting in healthcare research. BMC Medical Research Methodology. 2018;18(1):118.

3. Food Standard Agency. Food portion sizes. Third ed. London: Food Standard Agency 2002.

4. Zhou L, Zhang R, Yang H, Zhang S, Zhang Y, Li H, et al. Association of plant-based diets with total and cause-specific mortality across socioeconomic deprivation level: a large prospective cohort. European Journal of Nutrition. 2024;63(3):835-46.

5. Thompson AS, Candussi CJ, Tresserra-Rimbau A, Jennings A, Bondonno NP, Hill C, et al. A healthful plant-based diet is associated with lower type 2 diabetes risk via improved metabolic state and organ function: A prospective cohort study. Diabetes & Metabolism. 2024;50(1):101499.

6. Heianza Y, Zhou T, Sun D, Hu FB, Qi L. Healthful plant-based dietary patterns, genetic risk of obesity, and cardiovascular risk in the UK biobank study. Clinical Nutrition. 2021;40(7):4694-701.

7. Heianza Y, Zhou T, Sun D, Hu FB, Manson JE, Qi L. Genetic susceptibility, plant-based dietary patterns, and risk of cardiovascular disease. The American journal of clinical nutrition. 2020;112(1):220-8.

8. Liu F, Lv Y, Peng Y, Qiao Y, Wang P, Si C, et al. Plant-based dietary patterns, genetic predisposition and risk of colorectal cancer: a prospective study from the UK Biobank. Journal of Translational Medicine. 2023;21(1):669.

9. Shang X, Liu J, Zhu Z, Zhang X, Huang Y, Liu S, et al. Healthy dietary patterns and the risk of individual chronic diseases in community-dwelling adults. Nature Communications. 2023;14(1):6704.

10. VanderWeele TJ. Causal mediation analysis with survival data. Epidemiology. 2011;22(4):582-5.
